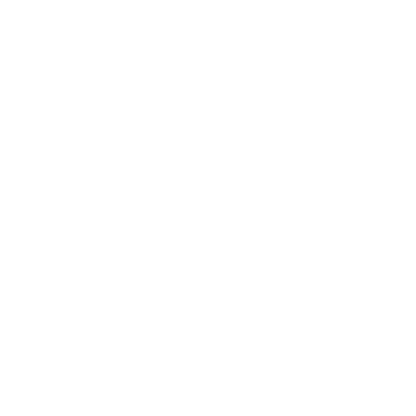# Correspondence: Strongly-driven Re+CO_2_ redox reaction at high-pressure and high-temperature

**DOI:** 10.1038/ncomms13647

**Published:** 2016-11-29

**Authors:** D. Santamaria-Perez, C. McGuire, A. Makhluf, A. Kavner, R. Chuliá-Jordan, J. L. Jorda, F. Rey, J. Pellicer-Porres, D. Martinez-García, P. Rodriguez-Hernández, A. Muñoz

**Affiliations:** 1 Earth, Planetary and Space Sciences Department, University of California Los Angeles, Los Angeles, California 951567, USA; 2 MALTA-Departamento de Física Aplicada-ICMUV, Universitat de València, 46100 Valencia, Spain; 3 Instituto de Tecnologia Quimica, Universitat Politècnica de València-CSIC, 46022 Valencia, Spain; 4 MALTA-Departamento de Física, Instituto Univ. de Materiales y Nanotecnología, Universidad de La Laguna, 38207 La Laguna, Tenerife, Spain

The discovery of non-molecular carbon dioxide phases under high pressure and temperature conditions with carbon tetrahedrally coordinated by oxygen atoms^1^,^2^,^3^ has shown that the high-density phase diagram of this important substance presents remarkable analogies with those of other group IV oxides. These results have triggered a variety of experimental studies aiming to explore the high-pressure high-temperature phase diagram of the CO_2_–SiO_2_ system, with a goal to find potentially stable compounds. Thus, although these oxides were traditionally perceived as being incompatible due to distinctive chemical behaviour, recent high-pressure high-temperature experiments have radically altered this view^4^,^5^.

In a recent paper, Santoro *et al.*^5^ reported the formation of a Si_0.4_C_0.6_O_2_ solid solution with a cristobalite-type structure after heating a mixture of a CO_2_-filled microporous silica polymorph, silicalite and CO_2_ in excess of 4,000 K while compressed at 16–22 GPa. We argue here that the authors could have misidentified this phase since the metallic rhenium observed in their X-ray diffraction patterns would have reacted with CO_2_ at 1,600 K to form β-ReO_2_ rhenium oxide.

In ref. 5, the authors synthesized the crystalline Si_0.4_C_0.6_O_2_ solid solution at pressures corresponding to a compromise between the respective stabilities of 3- and 4-fold coordination in CO_2_ (*P*>40 GPa) and 4- and 6-fold coordination in SiO_2_ (*P*>7 GPa), and temperatures where both pure SiO_2_ and CO_2_ are liquid. For these experiments, the SiO_2_:CO_2_ mixture located in sample chamber ∼80 μm in diameter was heated with a CO_2_ laser (*λ*=10.6 μm) with a beam focal spot of ∼30–40 μm, and the resulting compound was sampled using synchrotron X-ray radiation with a nominal 2 μm spot size.[Fig f1] Figure 2 of their paper shows the X-ray diffraction pattern at 7 GPa and room temperature, where the most intense diffraction peaks correspond to the new phase and Re from the gasket. Peaks of the new phase were indexed using a tetragonal unit cell and the systematic absences were consistent with the P4_1_2_1_2 space group, leading the authors to assign the α-cristobalite structure. The reported X-ray diffraction intensities do not correspond to randomly oriented powder so only peak positions and not relative intensities were used to infer the structure. The average formula of Si_0.4_C_0.6_O_2_ was consistent with X-ray diffraction and Raman spectroscopy results. This solid solution based on [CO_4_] tetrahedra was stable after quenching P-T down to ambient conditions.

We have recently studied the high-pressure chemistry of the CO_2_–SiO_2_ system up to 50 GPa and 2,400 K using double-sided laser heating in diamond-anvil cells, while characterizing the samples *in situ* by means of synchrotron-based X-ray diffraction. We performed eight independent runs of samples consisting of gas-loaded CO_2_, a CO_2_-filled zeolite (ITQ-29 (ref. 6) or silicalite-1F (ref. 7)) and a metallic heater, which acts as absorber of the diode pumped 1.064 μm laser. Heaters were pre-compressed pellets or thin coarse-grained disks of one of the following metals: Pt, Re or Au. After every heating run, we characterized the sample at high pressures and ambient temperatures performing a two-dimensional X-ray diffraction map of the pressure chamber traversing the laser-created hotspot.

Our results show that no chemical reaction occurs in the sample chamber at these high pressures and temperatures when gold or platinum were used as internal thermal couplers, neither between CO_2_ and SiO_2_, nor between these materials and the metallic heater. In these experiments, CO_2_-filled silica progressively amorphizises with pressure at ambient temperature, but crystalline Bragg peaks endures up to 24 GPa. Upon heating, at 1,300 K, the zeolites have transformed into the thermodynamically stable phase of SiO_2_ at the corresponding pressure, quartz for *P*<8 GPa and stishovite for *P*>8 GPa. Therefore, the supposed advantage of using zeolites to maximize surface chemical reactivity due to the large effective interaction area between the framework SiO_2_ and the confined CO_2_ is restricted to temperatures below 1,300 K. Our data show a progressive transformation from the zeolite, silicalite or ITQ-29 to stishovite above 16 GPa with increasing temperature. No other phase was identified in the scanned 300–1,300 K temperature gradient. CO_2_-I, -II, -III, -IV and -V phases were observed at different P-T conditions, the latter one being metastable during most of the downstroke pressure process.

When Re was used as a heater, a new phase was synthesized at pressures of 8 and 24 GPa and temperatures of 1,500–1,600 K. A more in-depth manuscript on the reactivity of transition metals and CO_2_ has been recently published^8^. This new phase coexists with stishovite, and it is present up to 48 GPa and also during the entire decompression process down to ambient conditions. Bragg peaks of the new phase are located at *d*-spacings similar to those of the cristobalite-structured Si_0.4_C_0.6_O_2_ compound reported by Santoro *et al.*^5^ This is illustrated in Fig. 1, where the theoretically calculated X-ray diffraction patterns of Santoro's hypothesized α-cristobalite Si_0.4_C_0.6_O_2_ and the new phase we observed are depicted together. However, a careful indexing of 16 peaks suggests they are better fit by an orthorhombic structure instead of the tetragonal space group of the reported cristobalite structure. The diffraction peaks of our recovered sample were indexed using an orthorhombic cell with lattice parameters: *a*=4.809(2), *b*=5.640(7) and *c*=4.599(2) Å, and a unit-cell volume of 124.75(12) Å^3^. This structure is consistent with the Pbcn structure for β-ReO_2_ reported by Magneli^9^.

The Rietveld refinement of X-ray diffraction pattern of our recovered sample shows that the Bragg peak intensities, systematic absences and unit-cell dimensions all show excellent agreement with those predicted theoretically for the Pbcn β-ReO_2_ structure (Fig. 2)^8^.

An additional experiment confirming the chemical reactivity between CO_2_ and Re was performed at 15 GPa, using only Re metal loaded in a diamond-anvil cell sample chamber with CO_2_. This reaction is accompanied by a significant drop in pressure. Additionally, Raman spectra of experiments where only these two materials were loaded in the sample show, at 21 GPa and room temperature, a broad band centred at 1,671 cm^−1^, which corresponds to vibrations in the graphene planes and is often called the ‘G band'. Therefore, we hypothesize that both we and Santoro *et al.* are observing the reaction: Re+CO_2_=ReO_2_+C (reduced).

Our experiments reproduced the pressure conditions but fall short of achieving the high temperature conditions of Santoro *et al.*^5^ in the SiO_2_:CO_2_ system (2,200 K versus 4,000 K). However, a close inspection of the published X-ray diffraction pattern of the hypothetically synthesized cristobalite Si_0.4_C_0.6_O_2_ solid solution shows that the positions and intensities of the Bragg peaks can be explained by β-ReO_2_ structure. Note that the authors only performed a LeBail fitting of the pattern and that not all the peak positions were accurately fitted (see Figs 1 and 2a in ref. 5). For instance, the peak at 13.01° cannot be explained using the α-cristobalite model, whereas it corresponds to the (202) reflection of β-ReO_2_. Moreover, theoretically predicted reflections of significant intensity by a α-cristobalite model, such as (110) at 6.60°, (112) at 9.79°, (203) at 14.32° and (004) at 14.47°, were not observed, or the (102) at 8.60°, which is considerably shifted. In summary, the formation of rhenium oxide in their experiments is strongly supported by the observation of Re gasket peaks in their experimental X-ray diffraction pattern. We also note that as a high Z material, even a small amount of ReO_2_ may generate significant intensities on an X-ray diffraction pattern.

Our results demonstrate a strongly driven carbon dioxide reduction reaction in the presence of Re metal at high pressures and temperatures. It is important to stress that the experimental results reported here do not explain the Raman spectrum reported by Santoro *et al.*^5^ for the Si_0.4_C_0.6_O_2_ solid solution after temperature quenching. β-ReO_2_ Raman scattering is largely different^8^. The present results, however, raise substantial doubts on the phase assignment of the high-pressure high-temperature silicon carbon oxide phase.

## Figures and Tables

**Figure 1 f1:**
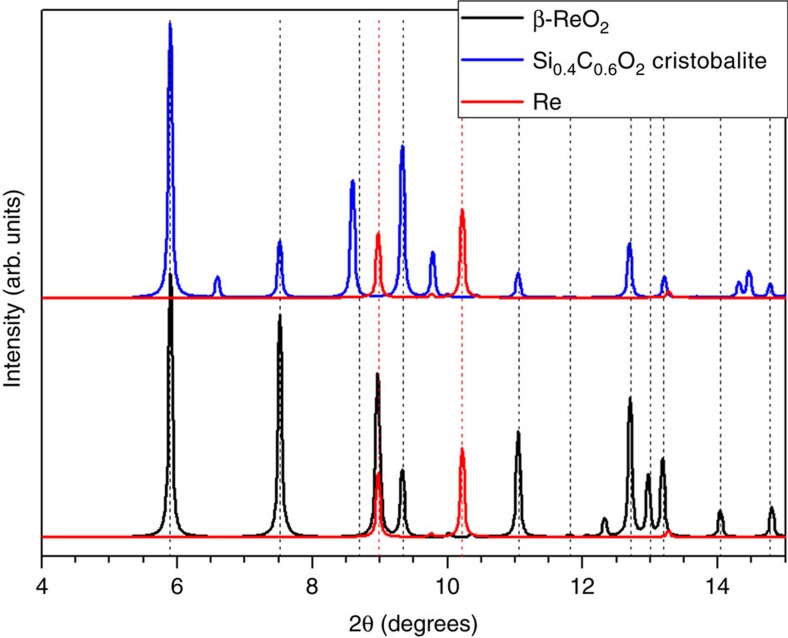
Calculated X-ray diffraction profiles of both the cristobalite Si_0.4_C_0.6_O_2_ solid solution and the ReO_2_ phases. Patterns were calculated to correspond to a pressure of 7 GPa, and *λ*=0.3738 Å. The calculated diffraction pattern for α-cristobalite Si_0.4_C_0.6_O_2_ is depicted in blue (lattice dimensions from ref. 5) and the calculated pattern for Pbcn β-ReO_2_ is represented in black (*a*=4.7735 Å, *b*=5.6047 Å and *c*=4.5755 Å). Calculated Re peaks are shown in red. Vertical dashed lines correspond to the positions of the X-ray diffraction peaks observed at 7 GPa by Santoro *et al.* (data obtained from a digitized version of Fig. 2 of ref. 5).

**Figure 2:**
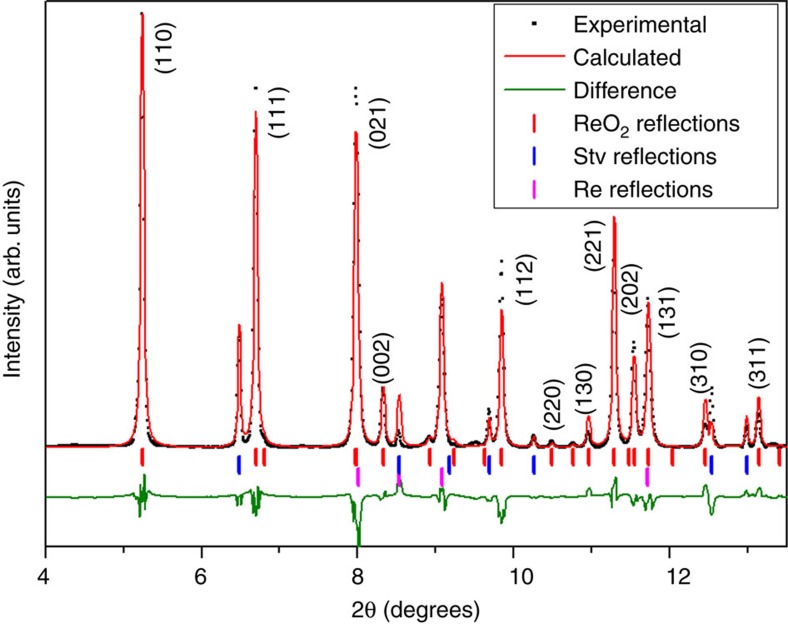
Rietveld refinement of the X-ray diffraction pattern at ambient conditions. This X-ray diffraction pattern corresponds to the recovered CO_2_:SiO_2_:Re sample after heating to 2,000 K and compressing to 48 GPa (*λ*=0.3344 Å). Reflections correspond to β-ReO_2_, Re and SiO_2_ stishovite. Experimental data are depicted as scattered black squares, and calculated and full difference X-ray diffraction profiles are represented as red and green lines, respectively. Refined lattice parameters are in the text, refined atomic coordinates are Re (4c: 0, 0.113(1), 0.25) and O (8d: 0.237(3), 0.362(2), 0.397(2)).

**Figure:**